# TILLING-by-Sequencing^+^ Reveals the Role of Novel Fatty Acid Desaturases (GmFAD2-2s) in Increasing Soybean Seed Oleic Acid Content

**DOI:** 10.3390/cells10051245

**Published:** 2021-05-19

**Authors:** Naoufal Lakhssassi, Valéria Stefania Lopes-Caitar, Dounya Knizia, Mallory A. Cullen, Oussama Badad, Abdelhalim El Baze, Zhou Zhou, Mohamed G. Embaby, Jonas Meksem, Aicha Lakhssassi, Pengyin Chen, Amer AbuGhazaleh, Tri D. Vuong, Henry T. Nguyen, Tarek Hewezi, Khalid Meksem

**Affiliations:** 1 Department of Plant, Soil and Agricultural Systems, Southern Illinois University, Carbondale, IL 62901, USA; naoufal.lakhssassi@siu.edu (N.L.); dounya.knizia@siu.edu (D.K.); mallory.cullen@siu.edu (M.A.C.); oussama.badad@gmail.com (O.B.); abdelhalim.elbaze@siu.edu (A.E.B.); zhou1228@siu.edu (Z.Z.); 2 Department of Plant Sciences, University of Tennessee, Knoxville, TN 37996, USA; vcaitar@utk.edu (V.S.L.-C.); thewezi@utk.edu (T.H.); 3 Department of Animal Science, Food, and Nutrition, Southern Illinois University, Carbondale, IL 62901, USA; mohamed.embaby@siu.edu (M.G.E.); aabugha@siu.edu (A.A.); 4 Trinity College of Arts and Sciences, Duke University, Durham, NC 27708, USA; jonas.meksem@duke.edu; 5 Faculty of Sciences and Technologies, University of Lorraine, 54506 Nancy, France; aicha.lakhssassi@gmail.com; 6 Division of Plant Sciences, University of Missouri, Columbia, MO 65211, USA; Chenpe@missouri.edu (P.C.); vuongt@missouri.edu (T.D.V.); nguyenhenry@missouri.edu (H.T.N.)

**Keywords:** high oleic acid, alternative fatty acid pathway, *GmFAD2-2* subfamily, TILLING-by-Sequencing^+^, subfunctionalization, neofunctionalization

## Abstract

Soybean is the second largest source of oil worldwide. Developing soybean varieties with high levels of oleic acid is a primary goal of the soybean breeders and industry. Edible oils containing high level of oleic acid and low level of linoleic acid are considered with higher oxidative stability and can be used as a natural antioxidant in food stability. All developed high oleic acid soybeans carry two alleles; *GmFAD2-1A* and *GmFAD2-1B*. However, when planted in cold soil, a possible reduction in seed germination was reported when high seed oleic acid derived from *GmFAD2-1* alleles were used. Besides the soybean fatty acid desaturase (*GmFAD2-1*) subfamily, the *GmFAD2-2* subfamily is composed of five members, including *GmFAD2-2A*, *GmFAD2-2B*, *GmFAD2-2C*, *GmFAD2-2D*, and *GmFAD2-2E*. Segmental duplication of *GmFAD2-1A*/*GmFAD2-1B*, *GmFAD2-2A/GmFAD2-2C*, *GmFAD2-2A/GmFAD2-2D*, and *GmFAD2-2D/GmFAD2-2C* have occurred about 10.65, 27.04, 100.81, and 106.55 Mya, respectively. Using TILLING-by-Sequencing+ technology, we successfully identified 12, 8, 10, 9, and 19 EMS mutants at the *GmFAD2-2A*, *GmFAD2-2B*, *GmFAD2-2C*, *GmFAD2-2D*, and *GmFAD2-2E* genes, respectively. Functional analyses of newly identified mutants revealed unprecedented role of the five *GmFAD2-2A*, *GmFAD2-2B*, *GmFAD2-2C*, *GmFAD2-2D*, and *GmFAD2-2E* members in controlling the seed oleic acid content. Most importantly, unlike *GmFAD2-1* members, subcellular localization revealed that members of the *GmFAD2-2* subfamily showed a cytoplasmic localization, which may suggest the presence of an alternative fatty acid desaturase pathway in soybean for converting oleic acid content without substantially altering the traditional plastidial/ER fatty acid production.

## 1. Introduction

Soybean oil is the one of the most consumed vegetable oils worldwide. Soybean oil’s utilization is determined by its fatty acid composition. Usually, the content of oleic acid (18:1, ω-9) in soybean oil is about 18–20% [1]. Consumption of oil with high oleic acid content is desirable because this monounsaturated fatty acid improves shelf life and reduces the need for hydrogenation [2]. Additionally, oil high in oleic acid and low in saturated fatty acids are desired by the biodiesel industry, in order to improve the oxidative stability while increasing cold flow [3].

In plants, mutations can be artificially induced by mutagenic agents and their utilization for production of new superior varieties of species from the traditional variety [4]. Genetic modification of the fatty acid composition of soybean oil is an important goal to improving soybean breeding for better oil traits [5,6,7,8]. While traditional breeding may take several years to achieve traits of interest, mutation breeding is the most useful and vital technology for soybean production. Selection of effective and efficient mutagens is very essential for recovering a high frequency of desirable mutants [9].

Mutations within the *GmFAD2-1A* and *GmFAD2-1B* genes encoding fatty acid desaturases (omega-6 FAD; EC 1.3.1.35) in soybeans was used to produce high oleic acid soybean germplasm [8,10]. However, it has been reported that high seed oleic acid derived from *GmFAD2-1* may have a possible reduction in seed germination when planted in cold soil [5,11,12,13]. The soybean community and industry are making tremendous efforts to determine the optimum allele combinations to produce environmentally stable high oleic/low linolenic acid soybean varieties for US soybean production environments (USB# 1720-162-0109). Therefore, looking for new alternatives to increase soybean seed oleic acid content while maintaining good yield is considered as high priority for the soybean industry. The fatty acid desaturase-2 enzyme (FAD2) is responsible for the conversion of oleic acid to linoleic acid in the developing soybean seeds by introducing a double bond at the Δ12 position of oleic acid [14,15,16]. This mono-unsaturated fatty acid contains only one double bond in its carbon chain. The high polyunsaturated fatty acid content in soybean oil exhibits low oxidative stability and must be hydrogenated for many applications, but such process introduces trans fats that cause a number of health problems in humans [17]. Elevated oleic acid content in soybean oil improves oxidative stability and shelf life to avoid hydrogenation and is considered healthier for human consumption [2]. Edible oils containing high level of oleic acid and low level of linoleic acid are considered with higher oxidative stability and can be used as a natural antioxidant in food stability [18,19,20,21]. The traditional *GmFAD2-1A* and *GmFAD2-B* genes were well characterized for their role in unsaturated fatty acid biosynthesis. However, members of the *GmFAD2-2* subfamily reported earlier [8] were not characterized yet. Initially, the expression of the two microsomal *GmFAD2-1* desaturases has been mainly detected in developing soybean seeds, therefore, *GmFAD2-1A* and *GmFAD2-1B* were regarded as the best candidate genes to develop soybean lines with elevated oleic acid content [22]. However, members of the other *GmFAD2-2* gene subfamily were poorly characterized for their role in the fatty acid pathway. The *GmFAD2-2* gene family in soybean is composed of five members including *GmFAD2-2A* (Glyma.19G147300), *GmFAD2-2B* (Glyma.19G147400), and *GmFAD2-2C* (Glyma.03G144500), *GmFAD2-2D* (Glyma.09G111900) and *GmFAD2-2E* (Glyma.15G195200) [8]. Using CRISPR/Cas9, it has been shown recently that *GmFAD2-1A* and *GmFAD2-2A* mutants accumulate high levels of soybean seed oleic acid [23,24].

Conventional breeding and genetic engineering have been widely applied to produce soybeans with oleic acid content > 80% of the total oil. Although downregulation of *GmFAD2-1* genes can achieve the elevated levels of oleate through ribozyme-terminated antisense, restrictive regulations from foreign destinations hindered the potentials in exportation of these transgenic soybean [25]. Identification of mutations in *GmFAD2-1* genes via reverse genetic approaches appears to be a sustainable strategy to develop non-transgenic soybean with high oleic acid content [8,10]. Several soybean lines with more than 80% seed oleic acid content have been produced through combining *GmFAD2-1A* and *GmFAD2-1B* alleles (soybean diversity) [5,26,27]. Additionally, using targeted mutagenesis with transcription activator-like effector nucleases (TALENs) in *GmFAD2-1* genes, nontransgenic high oleic acid content (80%) can be obtained [28].

The use of silencing and/or CRISPR techniques is another strategy that could be used to produce high oleic acid soybean lines [23,24]. However, although it has been accepted in the U.S. as nontransgenic approach, there is still a worldwide debate about the technology and concerns of the vectors used and transgenesis approach to produce and study the desired traits. Therefore, the characterization and availability of TILLING (Targeting Induced Local Lesions IN Genomes) mutants within the *GmFAD2-2* gene family members will not only elucidate and attribute a new function of these genes but will definitely speed up the use of these genes in breeding process.

Ethyl-methanesulfonate (EMS) mutagenesis alkylates DNA and can cause significant changes to the DNA bases of genes underlying agronomic traits such us oil biosynthesis. Producing EMS mutants with a single base pair mutation has shown to be a better strategy for developing lines with agronomically important traits without unintended poor agronomic characteristics [29,30]. Using EMS mutagenesis effects on DNA, significant changes to the genes and gene network underlying the oil profile in soybean can be achieved [7,8,10]. These changes are difficult to achieve using standard breeding techniques. In the current study, we developed a population of soybean EMS mutagenized lines and used TILLING by sequencing^+^ [29,30], to functionally characterize the five members of the *GmFAD2-2* subfamily in soybean.

## 2. Material and Methods

### 2.1. Development of an EMS Mutagenized Forrest Population

Forrest is a soybean cultivar that belongs to the Maturity Group V with resistance to several soybean pathogens including Soybean Cyst Nematode (SCN), Sudden Death Syndrome (SDS), and Reniform nematode, reasons why it was used originally as background to develop the EMS mutants. As it is a cultivar, Forrest could be easily used for breeding purposes to introgress the high oleic acid content trait into high-yielding lines without compromising their agronomic performance while transferring its package of resistance. 

The soybean *cv*. “Forrest” seed was used to develop an EMS mutagenized population (at 0.6% EMS) as shown earlier [29], and planted to harvest 4032 M2 families and then advanced to the M3 generation at the Horticulture Research Center at Southern Illinois University Carbondale, IL, USA. DNA from all 4032 M2 families has been used in the TILLING-by-sequencing^+^ platform to make the corresponding DNA pools. 

### 2.2. FAD2 Sequences and Phylogenetic Analysis

GmFAD2 sequences used in the phylogenetic analyses were retrieved from different databases including UniProt, NCBI, Soybase (W82.a2.v1), and Phytozome (v12.1). Sequences were identified by querying sequences from the seven members belonging to the two *GmFAD2* subfamilies against sequences from these databases using tblastn default parameters. Sequences from monocots, eudictos, and basal angiosperm with 90% identity/similarity and above were selected, in addition to other GmFAD2 homologs from plant primitive species including, algae, moss, and a lycophyte. The retrieved GmFAD2 sequences belong to sets of plants (48 in total) with fully sequenced genomes representing key positions in the angiosperm phylogenetic tree. Sequences were carefully inspected and corrected for annotation errors before use. Multiple sequence alignments of the retrieved GmFAD2s were performed using the MEGA4 software package [31] and the ClustalW sequence alignment tools. An unrooted phylogenetic tree was calculated with the neighbor-joining method [32]. Next, tree topology robustness was tested through bootstrap analysis of 1000 replicates.

### 2.3. Chromosomal Distribution and Synteny Analysis

The locations of the two fatty acid desaturase *GmFAD2-1* members and the five *GmFAD2-2* members in soybean and their corresponding chromosomes were obtained from the soybean genome annotation a2.v1 assembly (Williams 82 reference genome) available on the soybean database (SoyBase.org). Nonsynonymous (Ka) versus synonymous substitution (Ks) rates were calculated based on their values retrieved from the Plant Genome Duplication Database (PGDD). Based on the Ks values and the rate of 6.1 × 10^−9^ substitutions/site/year, the divergence time (T) was estimated using the following formula: Ks/(2 × 6.1 × 10^−9^) × 10^−6^ Mya [33].

### 2.4. Library Preparation, Probe Design and TILLING-by-Sequencing^+^

Genomic DNA samples from 42 “96-well plates” were pooled using a bidimensional scheme as shown recently [29]. Forty-four probes were constructed to amplify the whole gene region of *GmFAD2-1A* with up to 99.6% coverage, 77 probes were constructed to amplify the whole gene region of *GmFAD2-1B* with up to 98.4% coverage, 18 probes were constructed to amplify the whole gene region of *GmFAD2-2A* with up to 99.7% coverage, 58 probes were constructed to amplify the whole gene region of *GmFAD2-2B* with up to 98.8% coverage, 47 probes were constructed to amplify the whole gene region of *GmFAD2-2C* with up to 99.8% coverage, 37 probes were constructed to amplify the whole gene region of *GmFAD2-2D* with up to 98.9% coverage, and the *GmFAD2-2E* gene was covered by 20 probes with up to 99.8% coverage (Table S1). Probe synthesis, DNA library preparation, capture enrichment (using magnetic beads), and next generation sequencing (Illumina HiSeqX 2 × 150 bp) were carried out by Rapid Genomics LLC. (Gainesville, FL, USA) as described recently [30].

### 2.5. Variant Calling for Mutation Detection

The FASTQ raw reads were subjected to quality control using FastQC v0.11.9, trimming, and filtering of low-quality reads was performed using Trimmomatic V0.39 [34]. BWA v0.7.17 [35] was used to map clean reads to the Williams 82 reference genome. SAM tools v1.10 [36] were used to filter and sort the bam files to serve as an input for variant calling using Freebayes v1.0.0 [37] and CRISP v1.18.0 [38] The VCF files were filtered by VCF tools v0.1.16 [39] and visualized in IGV v 2.9.2 [40].

### 2.6. Mutation Density Evaluation

The mutation density is estimated using the formula as the total number of mutations divided by the total number of base pairs (amplicon size × individuals screened) as described earlier [41].

### 2.7. Analysis of Seed Fatty Acids

For EMS mutant lines, a five-seed sample taken from each M3 mutant line was placed in an envelope and manually crushed with a hammer. Fatty acid extraction procedure was carried out as previously described [42]. Five major fatty acid contents were measured from selected according to the two-step methylation procedure as described earlier [43].

### 2.8. Confirmation of the Mutants by SANGER Sequencing

The specific primers (Table S2) were designed to amplify the 7 fatty acid desaturases using the extracted DNAs as the templates with 38 cycles of PCR amplification at 94 °C for 30 s, 51 °C for 30 s, and 72 °C for 1 min. The PCR products were purified as shown earlier [8]. The purified PCR fragments were sequenced by GENEWIZ, LLC.

### 2.9. Homology Modeling of GmFAD2-2 Proteins and Mutational Analysis

Homology modeling of putative *Gm*FAD2-2A, *Gm*FAD2-2B, *Gm*FAD2-2C, *Gm*FAD2-2D, and *Gm*FAD2-2E protein structures was retrieved from bar.utoronto.ca database. The PDB template used to model these structures was 4zyo and the confidence value was 99.2. The 3D molecule data used in this study come from [44], Pfam domain data come from [45], and CDD feature hits come from [46]. Homology modeling shows an amino acid modeling rate of 43.98%, 77.28%, 78.32%, 78.75%, and 92.07% for *Gm*FAD2-2A, *Gm*FAD2-2B, *Gm*FAD2-2C, *Gm*FAD2-2D, and *Gm*FAD2-2E proteins, respectively. Mutation mapping and visualizations were performed using the UCSF Chimera package as shown earlier [47].

### 2.10. GmFAD2-1 and GmFAD2-2 Subcellular Localization and Cloning

The *Gm*FAD2-1A, *Gm*FAD2-1B, *Gm*FAD2-2A, *Gm*FAD2-2B, *Gm*FAD2-2C, *Gm*FAD2-2D, and *Gm*FAD2-2E coding sequences were amplified from “Forrest” cDNA using gene specific forward and reverse primers containing EcoRI and SalI restriction enzyme sites, respectively. The amplified PCR products were fused to the N-terminus of the yellow fluorescent protein (YFP) reporter gene in the pSAT6-EYFP-N1 vector. The fusion constructs were then verified by sequencing. Three micrograms of DNA for each plasmid were bombarded into onion epidermal cells as previously described [48]. The pSAT6-EYFP-N1 empty vector was used as a cytoplasmic control. Onion epidermal peels were incubated at 26 °C in the dark for at least 20 h. The subcellular localization of the fused proteins was visualized using the EVOS^®^ FL Auto Cell Imaging System (Life Technologies, Carlsbad, CA, USA). The subcellular localization experiment was repeated twice.

### 2.11. Analysis of Putative Cis-Elements at the GmFAD2-1 and GmFAD2-2 Promoters

Putative *cis*-elements in the upstream region (-2Kb upstream) of all 7 *GmFAD2-1* and *GmFAD2-2* gene members were searched using the programs PLACE, Plant PAN 2.0 and MatInspector [49,50,51]. Additional filtering was carried out based on motif score and redundant repeated motifs. Next, significant motifs were searched manually using PLACE for the putative role in plant development [49].

### 2.12. Statistical Analysis

All presented results were performed using JMP Pro 14 using the Student’s *t*-test for comparisons of means.

### 2.13. RNA-seq Library Preparation and Analysis

Four plant soybean tissues were used for RNA-seq including seed, leaf, root, flower and pods. Total RNA of each sample was extracted from 100 mg of frozen grounded samples using RNeasy QIAGEN KIT (Cat. No./ID: 74004). Total RNA was treated with DNase I (Invitrogen, Carlsbad, CA, USA). RNA-seq libraries preparation and sequencing were performed at Novogene INC. using Illumina NovaSeq 6000. The four libraries were multiplexed and sequenced in two different lanes generating 20 million raw pair end reads per sample (150bp). Quality assessment of sequenced reads was performed using fastqc, version 0.11.9 [34]. After removing the low-quality reads and adapters with trimmomatic, version V0.39 [34], the remaining high-quality reads were mapped to the soybean reference genome Wm82.a2.v1 using STAR, version v2.7.9 [52,53]. Uniquely mapped reads were counted using Python package HTseq v0.13.5 [54]. Read count normalization and differential gene expression analysis were conducted using the Deseq2 package v1.30.1 [55] integrated in the OmicsBox platform from BioBam (Valencia, Spain).

## 3. Results

### 3.1. FAD2 Duplication within the Soybean Genome

In soybeans, two *GmFAD2* subfamilies were previously reported [8]. Two members constitute the *GmFAD2-1* subfamily and five members belong to the *GmFAD2-2* subfamily. GmFAD2-1A and GmFAD2-1B are located on chromosomes Chrs.10 and 20, respectively. *GmFAD2-2A* and *GmFAD2-2B* are located in Tandem in Chr.19, whereas *GmFAD2-2C, GmFAD2-2D,* and *GmFAD2-2E* are located in Chr.03, 09, and 15, respectively (Figure S1). The four independent segmental duplicated blocks containing the genomic pairs *GmFAD2-2A/GmFAD2-2C*, *GmFAD2-2A/GmFAD2-2D*, *GmFAD2-2D/GmFAD2-2C*, and *GmFAD2-1A/GmFAD2-1B* were previously identified in ±100 kb duplicated regions centered around the *GmFAD2* genes [8]. However, *GmFAD2-2B* was not found to be a result of a segmental duplication. Synteny analysis from the current study suggests that *GmFAD2-2B* may be the result of a tandem duplication involving *GmFAD2-2A.*

The calculated ratios of nonsynonymous to synonymous substitutions (Ka/Ks) of the four *GmFAD2-2A/GmFAD2-2C* (Ka/Ks = 0.18), *GmFAD2-2A/GmFAD2-2D* (Ka/Ks = 0.12), *GmFAD2-2D/GmFAD2-2C* (Ka/Ks = 0.1), and *GmFAD2-1A/GmFAD2-1B* (Ka/Ks = 0.23) gene-pairs (chromosomal duplications) were less than 1, suggesting that their evolution may follow a purifying natural selection that could act on their coding sequences [56,57]. The duplication time of the five *GmFAD2* members was estimated to match the recent (*GmFAD2-1A/GmFAD2-1B* and *GmFAD2-2A/GmFAD2-2C*), and old (*GmFAD2-2A/GmFAD2-2D* and *GmFAD2-2D/GmFAD2-2C*) duplication events. The segmental duplication of *GmFAD2-1A/GmFAD2-1B* and *GmFAD2-2A/GmFAD2-2C* was calculated to have occurred about 10.65 and 27.04 Mya, while the segmental duplications of *GmFAD2-2A/GmFAD2-2D* and *GmFAD2-2D/GmFAD2-2C* may have occurred 100.81 and 106.55 Mya. These data suggest that the calculated duplication time of *GmFAD2-1A/GmFAD2-1B* and *GmFAD2-2A/GmFAD2-2C* was close to the suggested recent duplication event (~13 mya). The calculated duplication time of *GmFAD2-1A/GmFAD2-1B* and *GmFAD2-2A/GmFAD2-2C* may belong to the old duplication event (~59 mya) [58], which is consistent with the obtained soybean *GmFAD2* intragenome syntenic relationships calculated earlier using the Plant Genome Duplication Database [8].

### 3.2. Evolution of the GmFAD2 Gene Family

**Figure 1:**
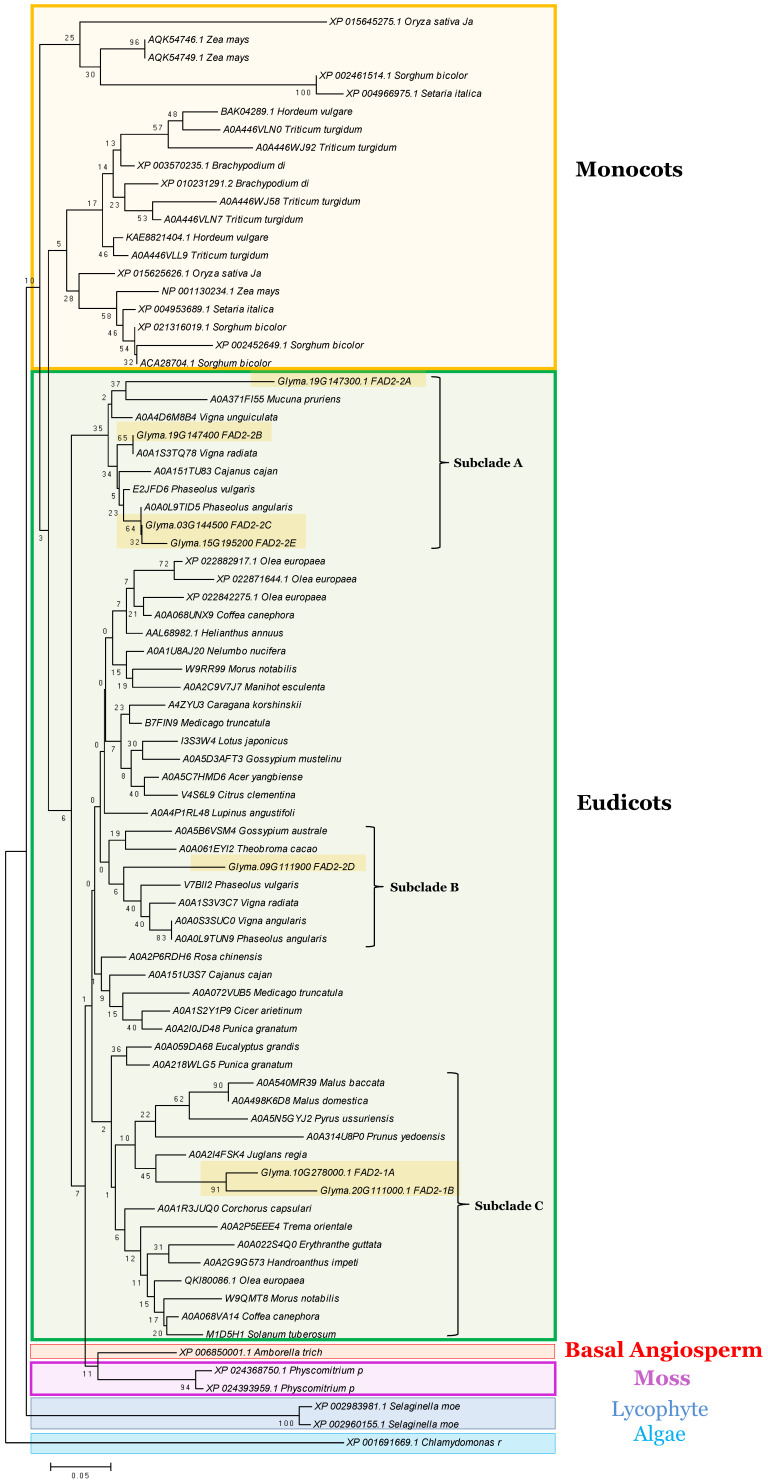
Phylogenetic tree of the fatty acid desaturase-2 from 48 sequenced plant species. FAD2 proteins identified in five model plants; *C. reinhardtii* (algae; green box), *P. patens* (moss), *S. moellendorfii* (lycophyte), *O. sativa* (monocot), and *M. truncatula* (eudicot leguminous), in addition to *G. max* (soybean) and other monocots and eudicots FAD2s were included in the analysis. The phylogenetic tree was generated using MEGA4 software package and the ClustalW algorithm and calculated using the neighbor-joining method. The tree bootstrap values are indicated at the nodes (*n* = 1000).

To understand the evolutionary relationships within the *GmFAD2* gene family, the seven *Gm*FAD2 protein members were aligned with orthologous protein sequences from 48 plant species, 7 monocots, 37 eudicots, and the most primitive plants including a basal angiosperm (*Amborella trichopoda*), a lycophyte (*Selaginella moellendorff*), a moss (*Physcomitrella patens*), and a chlorophyte (*Chlamydomonas reinhardtii*) (Figure 1).

Phylogenetic analysis separately grouped FAD2s from monocot, eudicot, a basal angiosperm, and the two primitive land species (mosses and lycophytes). The analysis shows that the ancestral FAD2 from the chlorophytic algae was outgrouped. These results demonstrate clearly that the fatty acid desaturase-2 followed the typical path of evolution, from aquatic to land plant species, being essential for plant survival. 

Within the eudicot clade, the analysis revealed the presence of three different subclades containing the seven FAD2 members. While the two GmFAD2-1A and GmFAD2-1B were found in the subclade (C) containing FAD2s from different tree species (Apple, Crab apple, Chinese pear, and English walnut), the other five GmFAD2-2 members were imbedded in two other different subclades containing FAD2s from several other leguminous. GmFAD2-2A, GmFAD2-2B, GmFAD2-2C, and GmFAD2-2E were grouped together in the subclade (A) and phylogenetically close to FAD2 leguminous including velvet bean, cowpea, mung bean, pigeon pea, common bean, and red mung beans (Figure 1). The GmFAD2-2D member was separately grouped in another subclade (B) containing other FAD2s from cacao tree, an endemic woody shrub, and FAD2 from other three leguminous (red mung bean, common bean, and mung bean).

### 3.3. Expression Analysis of GmFAD2 Gene Family

**Figure 2:**
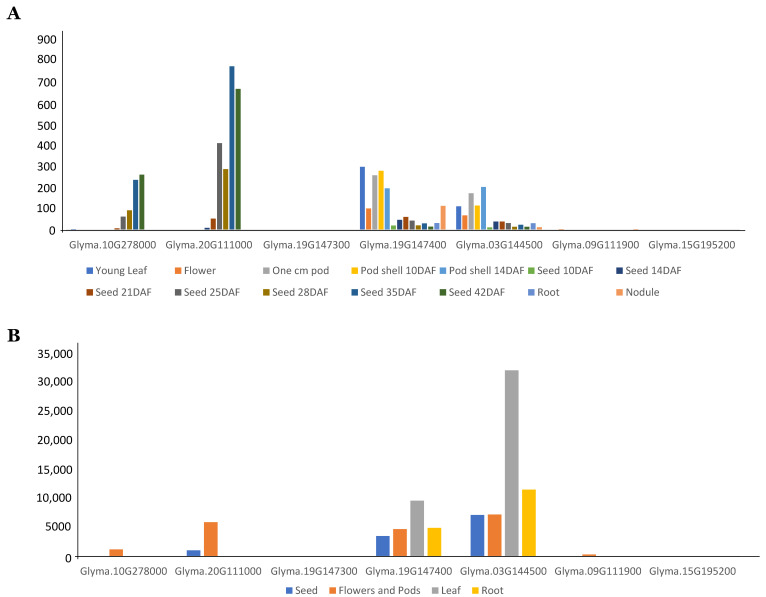
Expression pattern of soybean *GmFAD2-1* and *GmFAD2-2* gene members. (**A**) Expression of the seven GmFAD2 members in Williams 82 were retrieved from publicly available RNA-seq data from the SoyBase (http://www.soybase.org/soyseq), in addition to (**B**) the RNAseq data from the cultivar “Forrest”.

First, publicly available RNA-seq data of developing Williams 82 soybean seeds were examined. The expression pattern of all seven *GmFAD2* gene family members in the soybean reference genome Williams 82 was carried out in different tissues in order to investigate their specific evolutionary path. The two traditional *GmFAD2-1A* and *GmFAF2-1B* members showed the highest gene expression in seeds (at 35–42 DAF) (Figure 2). When comparing members of the *GmFAD2-2* gene family, both *GmFAD2-2B* and *GmFAD2-2C* transcripts were highly expressed in pod shell (at 10–14 DAF). *GmFAD2-2A, GmFAD2-2D,* and *GmFAD2-2E* showed the lowest expression.

To gain more insight into the expression of the seven *GmFAD2* members in soybean *cv.* Forrest (MG V), used to develop the mutagenized soybean population in this study, RNA-Seq analysis was carried out to check the expression levels of the *GmFAD2* members. RNA-Seq analysis showed that *GmFAD2-2B* and *GmFAD2-2C* transcripts were highly expressed than the traditional *GmFAD2-1* members in root and leaves (Figure 2). In seeds, flower, and pods, *GmFAD2-2B* and *GmFAD2-2C* together with *GmFAD2-1B* transcripts were more abundant than *GmFAD2-1A. GmFAD2-2A, GmFAD2-2D,* and *GmFAD2-2E* showed the lowest expression like in Williams 82 cultivar.

### 3.4. TILLING by Target Capture Sequencing 

**Figure 3:**
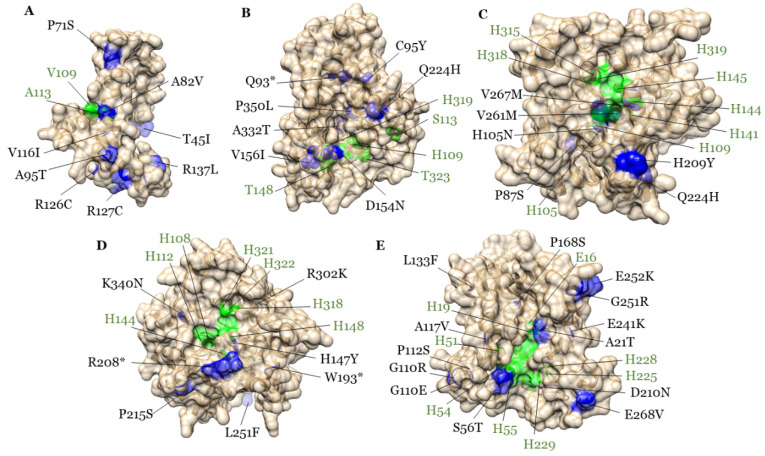
Homology modeling of the five GmFAD2-2 proteins and corresponding identified EMS mutations. Mutated residues on the five GmFAD2-2 proteins identified by Tilling-by-Sequencing^+^ were mapped on the five GmFAD2-2 protein homology models. Homology modeling of the GmFAD2-2A (**A**), GmFAD2-2B (**B**), GmFAD2-2C (**C**), GmFAD2-2D (**D**), and GmFAD2-2E (**E**) predicted proteins. The identified mutations and corresponding amino acid changes are shown on blue. Putative di-iron binding sites residues and substrate-binding pocket are shown in green.

To identify novel allelic variation within the *GmFAD2* gene family, a population of 4,032 EMS mutagenized soybeans was developed using the “Forrest” cultivar. Next, TILLING-by-Sequencing^+^ was used to identify several mutants from *GmFAD2-2A, GmFAD2-2B, GmFAD2-2C, GmFAD2-2D,* and *GmFAD2-2E*. Using this reverse genetic approach, we successfully identified 12 *GmFAD2-2A,* 8 *GmFAD2-2B,* 10 *GmFAD2-2C,* 9 *GmFAD2-2D,* and 19 *GmFAD2-2E* missense mutants (Figure 3).

### 3.5. Mutation Density of the “Forrest” EMS Mutagenized Soybean Population

The first soybean TILLING mutagenized populations were produced with a mutation density corresponding to ~1/140 kb and ~1/550 kb using EMS or N-nitroso-N-methylurea (NMU), respectively [41]. TILLING-by-Sequencing^+^ analysis of the seven fatty acid desaturase genes resulted in the identification of 441 SNP mutations and 16 InDels (Table 1). About 74% of mutations were the typical EMS mutations (G to A and C to T), while the other type of mutations account for about 26% of the total mutations. The mutation density is estimated to be ~1/155 kb, ~1/154 kb, ~1/128 kb, ~1/138, ~1/102, ~1/121, and ~1/67 kb for the *GmFAD2-1A, GmFAD2-1B, GmFAD2-2A, GmFAD2-2B, GmFAD2-2C, GmFAD2-2D,* and *GmFAD2-2E* genes, respectively. Within the coding regions, 50–70% of missense mutations, 1–8% of nonsense mutations, and 22–26% of silent mutations were obtained (Figure S1).

### 3.6. All Five GmFAD2-2s Are Involved in High Oleic Acid Content 

The identified EMS mutants were mapped on the five GmFAD2-2 protein models (Figure 3). The results showed that most of the mutations were mapped on key protein domains including the catalytic activity of the enzyme (di-iron center), homodimer interface, and/or substrate binding, suggesting that the isolated mutations may have a negative impact on protein activity and/or dimerization.

Most importantly, all isolated missense and nonsense *Gmfad2-2a, Gmfad2-2b, Gmfad2-2c, Gmfad2-2d,* and *Gmfad2-2e* mutants showed a significant increase in their seed oleic acid content when compared to the wild-type “Forrest” (Table 2). Mutations on *GmFAD2-2A, GmFAD2-2B, GmFAD2-2C, GmFAD2-2D,* and *GmFAD2-2E* resulted in an oleic acid increase with up to 31.9%, 28.1%, 29.6%, 32.7, and 35.7%, respectively. Our results showed that *GmFAD2-2A, GmFAD2-2B, GmFAD2-2C, GmFAD2-2D,* and *GmFAD2-2E* play an unprecedented role in unsaturated fatty acid biosynthesis in soybeans, suggesting that members of the *GmFAD2-2* subfamily may have been subfunctionalized in soybeans during the two whole genome duplication events.

### 3.7. Subcellular Localization of GmFAD2-1 and GmFAD2-2 Subfamily Members

**Figure 4:**
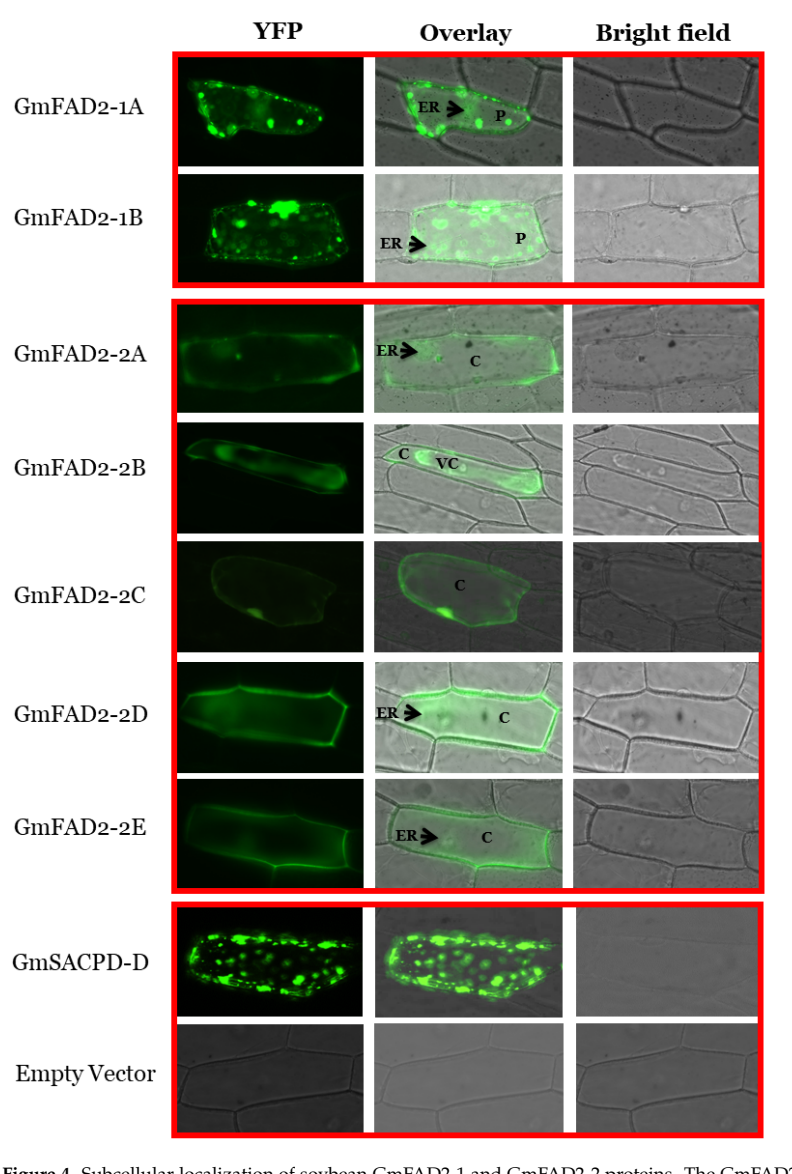
Subcellular localization of soybean GmFAD2-1 and GmFAD2-2 proteins. The GmFAD2 coding sequences from all seven members were fused to eYFP and delivered into onion epidermal cells using biolistic bombardment. GmFAD2-1A and GmFAD2-1B showed an endoplasmic reticulum (ER) and chloroplastic (P) localization pattern. GmFAD2-2A, GmFAD2-2D, and GmFAD2-2E showed a reticulum endoplasmic and cytosol (C) localization. GmFAD2-2C signal was found in the cytosol only. GmFAD2-2B shows a vacuole (VC) and cytoplasmic localization. The chloroplast-targeted GmSACPD-D was used as positive control [29]. Bar = 100 µM.

Although FAD2 genes were involved in converting oleic acid into linoleic acid, several subcellular localization patents of the FAD2 genes have been reported in different plant species. It has been shown that the fatty acid desaturase-2 from other plant species like *Arabidopsis thaliana* [59], *Artemisia sphaerocephala* [60], cucumber [61], and spinach [62] are located in the endoplasmic reticulum. Several studies have predicted the endoplasmic reticulum localization of the GmFAD2-1A and GmFAD2-1B proteins. However, up to date, no study has shown the subcellular localization of the two GmFAD2-1 and/or the five GmFAD2-2 subfamily members in soybean. In order to gain more insight into the function of the fatty acid desaturases-2 in soybeans, their subcellular localization was examined using YFP fusion in onion epidermal cells using biolistic bombardment. Onion epidermal cells expressing *GmFAD2-1s*:YFP fusion confirmed their localization in the endoplasmic reticulum, but also showed an interesting expression pattern in the chloroplasts (Figure 4). The accumulation of GmFAD2-1A and GmFAD2-1B in the endoplasmic reticulum and chloroplast is consistent with the role of this class of proteins in fatty acid desaturation reported earlier. *GmFAD2-2s*:YFP fusion showed a distinct pattern localization from *GmFAD2-1s*:YFP (Figure 4). While GmFAD2-2C signal was found only in the cytosol, GmFAD2-2B signal was located mainly in the vacuole, in addition to the cytosol. The other three members, GmFAD2-2A, GmFAD2-2D, and GmFAD2-2E showed a reticulum endoplasmic localization in addition to the presence of a cytosolic signal.

### 3.8. Analysis of Putative Cis-Elements in the Promoter Region of GmFAD2-1 and GmFAD2-2 Gene Members

**Figure 5:**
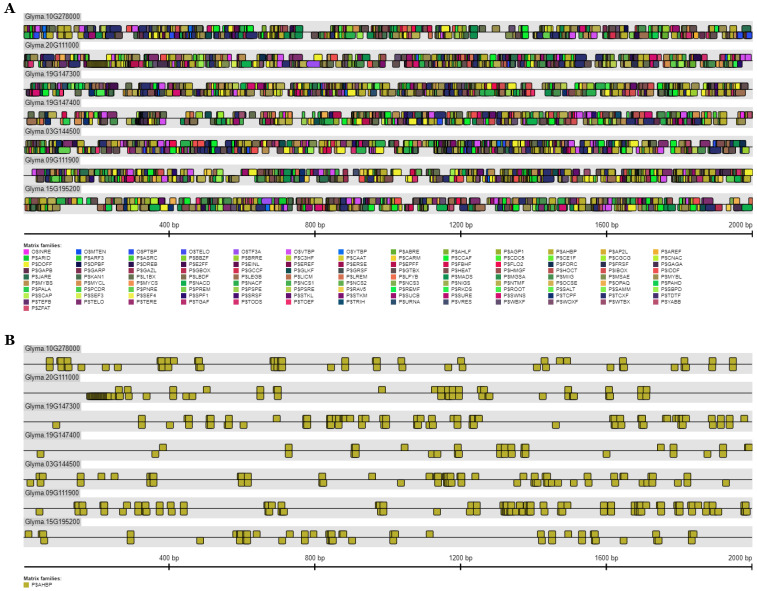
Analysis of putative *cis*-elements in the promoter region of the *GmFAD2-1* and *GmFAD2-2* gene members. (**A**) All identified *cis*-elements at the *GmFAD2-1* and *GmFAD2-2* promoter region (−2 Kb upstream). (**B**) Shows the conserved Arabidopsis homeobox protein domain (P$AHBP) that was shared between all *GmFAD2-1* and *GmFAD2-2* subfamily members with a total match that was significantly higher (459) when compared to the other *cis*-elements shown in (**A**).

The analysis of putative *cis-*elements in the promoter region (−2 Kb upstream) of the translation start codon of *GmFAD2-1* and *GmFAD2-2* gene members showed an enrichment of a *cis*-binding motifs for the Arabidopsis homeobox protein domain (Figure 5). The frequency of this *cis*-element was significantly higher (459) when compared to the other *cis-*elements that are present in the *GmFAD2*s promoter region (2.65–459 times higher) (Table S3). The promoter analysis shows that *GmFAD2-1A*, *GmFAD2-1B*, *GmFAD2-2A*, *GmFAD2-2B*, *GmFAD2-2C*, *GmFAD2-2D*, and *GmFAD2-2E* promoters contain 59, 64, 81, 34, 67, 102, and 52 Arabidopsis homeobox protein binding elements, respectively. An extremely high presence of the Arabidopsis homeobox protein binding element was also observed in *GmFAD2-1A* and *GmFAD2-1B* promoter regions (81 and 102, respectively). These data may suggest an involvement of Arabidopsis homeobox protein in the oleic acid biosynthesis. This is coherent with a previous study showing the involvement of a homeodomain transcription factor in lipid metabolism. In fact, overexpression of the epidermis-specific homeodomain-leucine zipper IV transcription factor Outer Cell Layer1 in maize identifies target genes involved in lipid metabolism and cuticle biosynthesis [63]. 

Additionally, the promoter analysis revealed the absence of the DNA-binding proteins with the plant specific TCP-domain in the GmFAD2-1 subfamily that was shared only between the five members of the GmFAD2-2 subfamily (Table S3). The presence of this *cis*-element within the *GmFAD2-2* subfamily only may be linked to specific mode of regulation taking into consideration the subcellular localization pattern that was different from the GmFAD2-1 subfamily.

## 4. Discussion

### 4.1. Involvement of the Five GmFAD2-2 Members in the Unsaturated Fatty Acid Pathway

**Figure 6:**
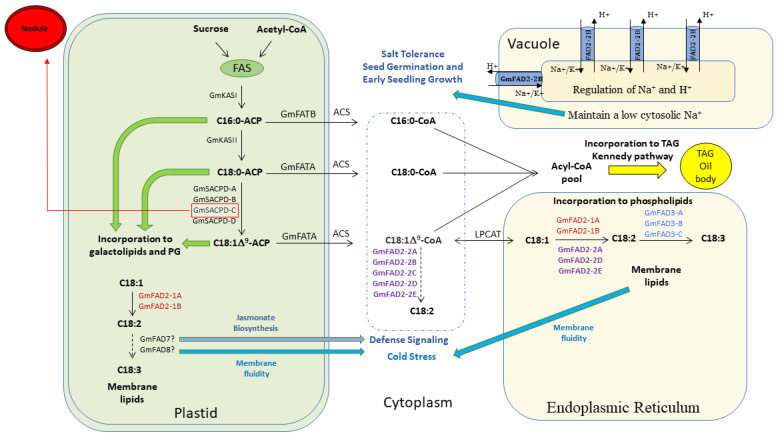
Cartoon deciphering the soybean seed fatty acid biosynthesis pathway. The seven *GmFAD2-1* and *GmFAD2-2* members are responsible for converting oleic acid (C18:0-ACP) to linoleic acid (C18:1Δ^9^-ACP) in the plastid (GmFAD2-1A and GmFAD2-1B), ER (GmFAD2-1A, GmFAD2-1B, GmFAD2-2A, GmFAD2-2D, and GmFAD2-2E), and cytoplasm (GmFAD2-2A, GmFAD2-2B, GmFAD2-2C, GmFAD2-2D, and GmFAD2-2E). GmFAD2-2B is located in the vacuole and is likely to play a role during seed germination and early seed growth by maintaining low cytosolic Na^+^ as described in Arabidopsis [65]. In bold are the five newly identified fatty acid desaturases involved in the unsaturated fatty acid biosynthesis. GmFAD2-2A, GmFAD2-2B, GmFAD2-2C, GmFAD2-2D, and GmFAD2-2E members present a good alternative for converting oleic acid content without substantially altering the traditional plastidial/ER fatty acid production pathway in soybean. The flux of fatty acids between the plastid and the ER occurs among different plant species [66]. A portion of C16:0-ACP, C18:0-ACP, and C18:1-ACP pool can be incorporated into phosphatidylglycerol (PG) and galactolipids in the plastid. A portion of the acyl-CoA moieties can be incorporated into triacylglycerol (TAG, the major lipid fraction in plant seed oils) biosynthesis in the seed via the Kennedy pathway. *GmFAD3* genes are differentially expressed during seed development or cold temperature exposure [67]. The *GmFAD7/8* promote C18:3 biosynthesis in the plastid, acting as precursors for the biosynthesis of Jasmonate and are expected to be involved in defense responses and biotic stress signaling [67]. GmSACPD-C is the nodule specific isoforms as shown earlier [29].

Several studies have investigated the role and function of the *FAD2* genes in several plant species. FAD2s were well studied and known for their roles in unsaturated fatty acid biosynthesis by converting oleic acid to linoleic acid. Traditionally, fatty acids are synthesized in plastid/endoplasmic reticulum [64]. In soybeans, members of the *GmFAD2-1* subfamily have been reported to increase seed oleic acid [5,8]. The endoplasmic reticulum subcellular localization of the traditional GmFAD2-1A and GmFAD2-1B subfamily members is consistent with the subcellular localization reported earlier in other plant species including shrub [60] and Arabidopsis [59]. In cucumber, while the retention signal of some fatty acid desaturases like CsFAD2 and CsFAD3 was found to target the endoplasmic reticulum, other fatty acid desaturases like CsFAD4, CsFAD5, CsFAD6, CsFAD7 and three CsFAB2s contained a predicted chloroplast signal peptide [61]. This is consistent with the chloroplastic localization of the GmFAD2-1A and GmFAD2-1B proteins shown in this study. EMS induced and spontaneous occurring mutations at the *GmFAD2-1A* and *GmFAD2-1B* genes were widely used in the soybean breeding programs to increase seed oleic acid content up to 85% after combining the two alleles to generate double *GmFAD2-1A*/*GmFAD2-1B* mutants [26,27]. However, very little is known about the role of the other members of the *GmFAD2-2* subfamily. Using TILLING-by-Sequencing^+^, we successfully identified several mutations within the five members of the *GmFAD2-2* subfamily and showed for the first time their involvement in increasing seed oleic acid content (Figure 6). Interestingly, in addition to their subcellular cytoplasmic localization, GmFAD2-2A, GmFAD2-2D, and GmFAD2-2E members have shown an endoplasmic reticulum localization, while GmFAD2-2B showed a clear vacuole localization (Figure 6). Although fatty acids were shown to be synthesized in Plastid/endoplasmic reticulum (ER), it has been reported that this synthesis can also happen directly from malonyl-CoA in the cytoplasm without substantially altering plastidial/ER fatty acid production, known as “de novo pathway” [64]. Therefore, the presence of such pathway in soybean involving members of the *GmFAD2-2* subfamily may have a positive impact on developing soybean lines with increased fatty acid and/or high very long chain polyunsaturated fatty acids that present several benefits for human health (Figure 6). 

Protein homology modeling analysis revealed that most of the mutations were mapped on key protein domains including the catalytic activity of the enzyme (di-iron center), homodimer interface, and/or substrate binding, suggesting that the isolated mutations may have a negative impact on the protein activity and/or dimerization. This could be explained by the fact that mutations at the fatty acid desaturase2-2 proteins (GmFAD2-2) failed to convert seed oleic acid into linoleic acid or possess very low enzymatic activity. Since enzymatic reactions are commonly reversible, is it possible that mutated desaturases are capable of converting linoleic acid (generated by normal desaturases) into oleic acid. This may suggest that it is possible that mutated desaturases not only lose original enzymatic activity, but also may catalyze the reverse reaction impacting positively the seed oleic acid content as seen in all the isolated GmFAD2-2 TILLING mutants.

Moreover, the accumulation of GmFAD2-2B in the cytosol and vacuole may suggest another role in controlling fatty acid desaturation at the plasma membrane and controlling ion exchange activity impacting the fluidization of membrane lipids, being essential for abiotic stress tolerance and early seedling growth [65] (Figure 6). In fact, the Arabidopsis fatty acid desaturase AtFAD2 was shown to play an essential role in plant resistance to salt stress by controlling the Na^+^/H^+^ exchange activity. The Arabidopsis AtFAD2 mediates a high-level of vacuolar and plasma membrane fatty acid desaturation. Plants maintaining a high Na^+^/H^+^ ratio in the cytosol show a high tolerance to soil salinity, a major abiotic stress that results in considerable crop yield losses worldwide [68]. Additionally, it is also essential for the proper function of membrane attached Na^+^/H^+^ exchangers to maintain a low cytosolic Na^+^ concentration for salt tolerance during seed germination and early seedling growth in Arabidopsis. The observed differences in subcellular localization may suggest that the GmFAD2-2B member underwent a process of neofunctionalization within the soybean genome, unlike the rest of the GmFAD2-2 members. Our subcellular localization data of the two GmFAD2 subfamily members is congruent with the evolution pattern shown earlier, which suggests that their evolution pattern may dictate their subcellular localization. 

Up to date, soybean geneticists and breeders have heavily used induced mutations (EMS and fast neutron), natural variations and/or genetic engineering approaches to increase oleic acid content up to 85%. TALEN and CRISPR technologies were recently used to create targeted mutations based on *GmFAD2-1A/GmFAD2-1B* genes. The available high oleic acid soybeans based on *GmFAD2-1A/GmFAD2-1B* alleles (plastidial/ER fatty acid production) present affected germination in cold soil [13]. Loss of function of the GmFAD2-1A and GmFAD2-1B may affect the incorporation of fatty acids into phospholipids in the endoplasmic reticulum impacting membrane lipids and membrane fluidity; therefore, affecting cold stress tolerance and fatty acid stability of these lines [66]. Thus, the discovery of new fatty acid desaturases impacting positively the seed oleic acid content without disturbing the plastid/ER pathway and subsequent incorporation to phospholipids is extremely beneficial to develop an alternate strategy to improve seed oleic acid in soybean and their commercialization (Figure 6). 

### 4.2. Subfunctionalization of GmFAD2-2 Gene Family during Whole Genome Duplication 

The soybean genome has been diversified due to the presence of two different large-scale duplication events (~13 and 59 million years ago) [58], resulting in a paleopolyploid genome where three quarters of the genes are present in multiple copies [69], impacting the development of important agronomic traits [70,71]. As a consequence of these two duplication events, the two *GmFAD2-1* and *GmFAD2-2* subfamilies resulted in seven *GmFAD2* members that derived from three independent syntenic duplicated genomic regions and one tandem duplication. These data may suggest the existence of a common *FAD2* ancestor. The identification of a single *FAD2* gene in *C. reinhardtii* in addition to the evolutionary conservation of the *FAD2* proteins among soybeans from phylogenetically separated species further support this feature. Additionally, the fact that all five members of the *GmFAD2-2* subfamily are involved in the unsaturated fatty acid biosynthesis, similar to the *GmFAD2-1* subfamily, points to the presence of a subfunctionalization event of the *GmFAD2* gene family, which may be most probably the result of successive duplications of an ancestral *FAD2*, leading to the enhancement of soybean oil biosynthesis. Like the GmFAD2-1 subfamily, stacking more *GmFAD2-2* members is expected to provide additive effect leading to increasing the seed oleic acid content in soybean without the alteration of the plastidial/ER fatty acid production. The presence of subfunctionalization event has been reported earlier in soybeans. Two members of the Soluble NSF attachment proteins, the GmSNAP18 and GmSNAP11, have subfunctionalized to play a role in resistance to soybean cyst nematode [72], in addition to the four members of the Stearoyl-acyl carrier protein desaturases, which have been subfunctionalized to play a role in the fatty acid unsaturation by converting seed stearic acid to seed oleic acid. Furthermore, the observed substantial changes in *GmFAD2* gene expression may be most probably due to gene duplication and selection pressure imposed by environmental conditions. This may explain functional differences of the oleic acid and linoleic acid contents observed within the two *GmFAD2* gene subfamilies. Although the current study showed the potential of using members of the *GmFAD2-2* gene subfamily to develop soybean lines with increased seed oleic acid content, their specific role in the cytoplasm/plasma membrane needs to be further investigated.

## 5. Conclusions

Using a novel technology, TILLING-by-Sequencing^+^, we functionally characterized the five members of the *GmFAD2-2* subfamily. The identified mutations showed the presence of a positive impact on increasing soybean seed oleic acid content. Subcellular localization indicated that members of the two GmFAD2-2 subfamilies are located in cellular compartments different from those previously reported for the traditional GmFAD2-1s, suggesting the presence of an alternative pathway to convert oleic acid to linoleic acid in soybeans without substantially altering the traditional plastidial/ER fatty acid production. The isolated soybean TILLING mutants from this study can be used in soybean breeding programs to improve seed fatty acid composition trait.

## Figures and Tables

**Table 1:** SNP mutations, InDels, and mutation density for the *GmFAD2-1A*, *GmFAD2-1B, GmFAD2-2A*, *GmFAD2-2B*, *GmFAD2-2C*, *GmFAD2-2D*, and *GmFAD2-2E* genes.

Gene ID	Amplicon Size (bp)	Base Changes	Type of Base Changes			InDel	Mutation Density (Kb)	AA Changes	Missense Mutations	Nonsense Mutations	Silent Mutations
			G > A	C > T	Others						
Glyma.10G278000	1928	50	13	29	8	0	1/155	28	17	0	11
Glyma.20G111000	3403	89	22	29	38	3	1/154	25	11	1	13
Glyma.19G147300	889	28	13	12	3	1	1/128	13	9	1	3
Glyma.19G147400	2711	79	26	39	14	5	1/138	39	28	2	9
Glyma.03G144500	2210	87	29	35	23	3	1/102	28	15	1	12
Glyma.09G111900	1663	55	18	23	14	2	1/121	33	25	2	6
Glyma.15G195200	881	53	16	24	13	2	1/67	30	25	0	5
	**Total**	**441**	**137**	**191**	**113**	**16**		**196**	**130**	**7**	**59**

**Table 2:** All 58 isolated EMS *Gmfad2-2* TILLING mutants showing increase in the seed oleic acid content.

Gene ID	Plant ID	Nucleotide Change	Amino Acid Substitution	C16:0	C18:0	C18:1	C18:2	C18:3
**GmFAD2-2A** **Glyma.19g147300**	**F490**	G410T	R137L	10.4	3.5	**29.6**	**50.1**	6.4
	**F601**	G283A	A95T	9.6	4.8	**24.8**	54.3	6.4
	**F300**	C134T	T45I	10.7	4.1	**25.1**	54.0	6.1
	**F1410**	C38A	P13H	10.6	**5.1**	**27.8**	**50.9**	5.6
	**F960**	C91T	R31C	10.2	3.6	24.2	54.3	7.6
	**F935**	C103T	R35C	10.3	3.7	**25.1**	53.4	7.5
	**F239**	C211T	P71S	11.1	4.2	**26.8**	53.3	4.6
	**F1044**	C245T	A82V	10.0	**5.2**	**26.1**	54.1	4.6
	**F1202**	C331T	H11Y	9.4	4.1	**31.9**	**49.0**	5.5
	**F141**	G346A	V116I	10.8	**5.1**	**27.9**	**50.3**	5.8
	**F1581**	C376T	R126C	10.5	4.5	**28.6**	51.2	5.2
	**F1493**	C379T	R127C	9.7	3.4	**25.8**	54.5	6.6
**GmFAD2-2B** **Glyma.19g147400**	**F211**	G994A	A332T	10.7	3.9	**25.1**	52.0	8.4
	**F185**	C277T	Q93 *	9.6	3.5	**25.6**	**50.6**	10.7
	**F45**	G1118A	S373N	10.8	3.7	**26.3**	52.1	7.1
	**F1103**	G284A	C95Y	10.3	3.5	**28.1**	**51.3**	6.9
	**F1496**	G460A	D154N	9.7	**4.8**	**27.8**	**51.3**	6.4
	**F1562**	G466A	V156I	9.9	4.2	**27.3**	52.2	6.4
	**F253**	A672T	Q224H	10.1	4.2	**27.4**	52.7	5.6
	**F921**	C1049T	P350L	10.2	3.7	24.1	53.5	8.5
**GmFAD2-2C** **Glyma.03G144500**	**496**	C625T	H209Y	9.9	3.1	**28.3**	**50.5**	8.3
	**1106**	G1114A	E372K	10.2	4.2	**29.6**	**50.8**	5.3
	**468**	C88T	P30S	9.9	**5.6**	**28.2**	**50.0**	6.2
	**595**	G781A	V261M	10.4	**5.1**	**25.7**	53.5	5.3
	**F408**	G49A	E17K	10.3	4.3	**28.9**	**51.3**	5.2
	**F1222**	G175A	D59N	10.4	4.0	**25.2**	52.7	7.7
	**F1748**	C259T	P87S	10.3	4.7	**26.1**	**51.0**	7.9
	**F1111**	C313A	H105N	10.5	**5.3**	**25.5**	52.7	6.0
	**F58**	A672T	Q224H	10.2	**4.9**	24.4	53.7	6.9
	**F1165**	G799A	V267M	9.3	3.2	**27.9**	**50.2**	9.4
**GmFAD2-2D** **Glyma.09G111900**	**313**	C643T	P215S	11.0	**4.9**	**26.9**	**48.8**	8.4
	**81**	A622T	R208 *	10.3	**5.2**	**32.7**	**46.5**	5.2
	**369**	C751T	L251F	10.7	**5.0**	**25.3**	53.1	5.9
	**1436**	G1094T	C365F	10.4	**5.2**	**27.7**	**50.0**	6.7
	**F1381**	A1020T	K340N	10.1	4.1	**28.9**	**50.2**	6.7
	**F275**	G905A	R302K	10.5	**6.4**	**29.2**	**47.7**	6.2
	**F1117**	G579A	W193 *	10.6	4.4	**27.7**	**50.6**	6.7
	**F470**	G510A	W170 *	9.7	4.7	**30.5**	**48.1**	6.9
	**F1268**	C439T	H147Y	9.8	4.0	**26.0**	52.5	7.6
**GmFAD2-2E** **Glyma.15g195200**	**F1087**	G329A	G110E	10.3	3.8	**21.3**	55.7	8.9
	**F380**	C502T	P168S	9.9	4.2	**24.6**	53.5	7.7
	**F9**	C829T	P277S	10.9	4.3	**24.0**	53.6	7.3
	**F1215**	A803T	E268V	10.6	4.9	**29.9**	**48.4**	6.2
	**F602**	G754A	E252K	10.4	4.2	**25.2**	54.0	6.2
	**F1328**	G751A	G251R	10.2	4.7	**27.2**	52.4	5.5
	**F1285**	G721A	E241K	10.4	3.9	24.4	52.5	8.8
	**F1065**	C706T	H236Y	11.3	4.8	**29.8**	**47.6**	6.5
	**F226**	G628A	D210N	11.3	**5.0**	**33.7**	**44.9**	5.1
	**F69**	G626A	R209k	10.5	4.5	**25.9**	52.1	7.0
	**F1803**	G605A	G202E	12.3	**5.0**	**32.0**	**45.5**	5.2
	**F480**	T595A	W199R	10.9	4.5	**26.4**	51.6	6.6
	**F372**	C397T	L133F	10.5	3.6	**25.7**	53.9	6.3
	**F1180**	C350T	A117V	10.5	4.2	24.0	54.5	6.9
	**F123**	C334T	P112S	10.4	**5.3**	**28.6**	**50.4**	5.3
	**F238**	G328A	G110R	10.2	**6.3**	**27.2**	**50.5**	5.8
	**F1480**	C167T	S56F	9.8	4.2	**25.7**	53.0	7.3
	**F1711**	T166A	S56T	9.4	**5.1**	**35.7**	**43.5**	6.3
	**F1462**	G61A	A21T	9.7	3.8	**33.3**	**47.8**	5.4
	**FWT**			**11.6**	**3.32**	**18**	**54.5**	**6.19**

* TILLING *Gmfad2-2* nonsense mutants resulting in stop codons. Fatty acid content in bold shows the increased seed oleic acid and decreased linoleic acid content due to the presence of deleterious mutations affecting the GmFAD2-2 protein activity. Underline oleic acid content shows the highest seed oleic acid content that was obtained in each one of the five GmFAD2-2 members. Some *Gmfad2-2* mutants showed a significant increase in their seed stearic acid content (bold).

## Supplementary Materials

The following are available online at https://www.mdpi.com/article/10.3390/cells10051245/s1, Figure S1. Physical positions and gene structures corresponding to the seven *GmFAD2*s are shown. (A) Six chromosomes carry the *GmFAD2* gene family. Glyma.10G278000 (Chr10: 50,013,484–50,015,460); Glyma.20G111000 (Chr20: 35,315,629–35,319,062); Glyma.19G147300 (Chr19: 40,814,864–40,815,855); Glyma.19G147400 (Chr19: 40,819,961–40,824,925); Glyma.03G144500 (Chr03: 36,014,623–36,020,910), Glyma.09G111900 (Chr09: 22,174,486–22,179,275), Glyma.15G195200 (Chr15: 22,308,021–22,308,902). (B) Gene structures of soybean fatty acid desaturases from three gene families. The structures of 19 soybean fatty acid desaturases genes were plotted with yellow boxes representing exons (coding DNA sequence, CDS), black lines illustrating introns, and blue boxes indicating 5′-UTR and 3′-UTR regions. The size of gene structures could be measured by the scale in the unit of base pair (bp) at the bottom. The gene structure was drawn using the Gene Structure Display Server. Table S1. Designed *GmFAD2-1A*, *GmFAD2-1B*, *GmFAD2-2A*, *GmFAD2-2B*, *GmFAD2-2C*, *GmFAD2-2D*, and *GmFAD2-2E* probes used for TILLING by Target Capture Sequencing. Table S2. Primers used for target Sanger sequencing. Table S3. Summary of the identified *cis*-elements at the promoter region (−2 Kb upstream) of the translation start codon of *GmFAD2-1* and *GmFAD2-2* gene members showing an enrichment of the Arabidopsis homeobox protein domain within the seven *GmFAD2* members.
