# Supplementary material for: TILLING-by-Sequencing+ Reveals the Role of Novel Fatty Acid Desaturases (GmFAD2-2s) in Increasing Soybean Seed Oleic Acid Content
Source: Cells. 2021 May 19;10(5):1245. doi: 10.3390/cells10051245 (PMC8158723; doi:10.3390/cells10051245)
Supplement: Supplementary file 1 [file cells-10-01245-s001.zip › cells-1217875-supplementary.pdf]

TILLING-by-Sequencing$^+$ reveals the role of novel Fatty acid desaturases (*GmFAD2-2*s) in increasing soybean seed oleic acid content

Naoufal Lakhssassi[1], Valéria Stefania Lopes-Caitar[2†], Dounya Knizia[1†], Mallory A. Cullen[1†], Oussama Badad[1], Abdelhalim El Baze[1], Zhou Zhou[1], Mohamed G. Embaby[3], Jonas Meksem[4], Aicha Lakhssassi[5], Pengyin Chen[6], Amer AbuGhazaleh[3], Tri D. Vuong[6], Henry T. Nguyen[6], Tarek Hewezi[2], and Khalid Meksem[1*]

[1]Department of Plant, Soil and Agricultural Systems, Southern Illinois University, Carbondale, IL 62901, USA.

[2]Department of Plant Sciences, University of Tennessee, Knoxville, TN, 37996.

[3]Department of Animal Science, Food, and Nutrition, Southern Illinois University, Carbondale, IL 62901, USA.

[4]Trinity College of Arts and Sciences, Duke University, Durham, NC 27708.

[5]Faculty of Sciences and Technologies, University of Lorraine, 54506, Nancy, France.

[6]Division of Plant Sciences, University of Missouri, Columbia, MO 65211, USA.

*Corresponding author, email address: meksem@siu.edu.

† These authors contributed equally to the work.

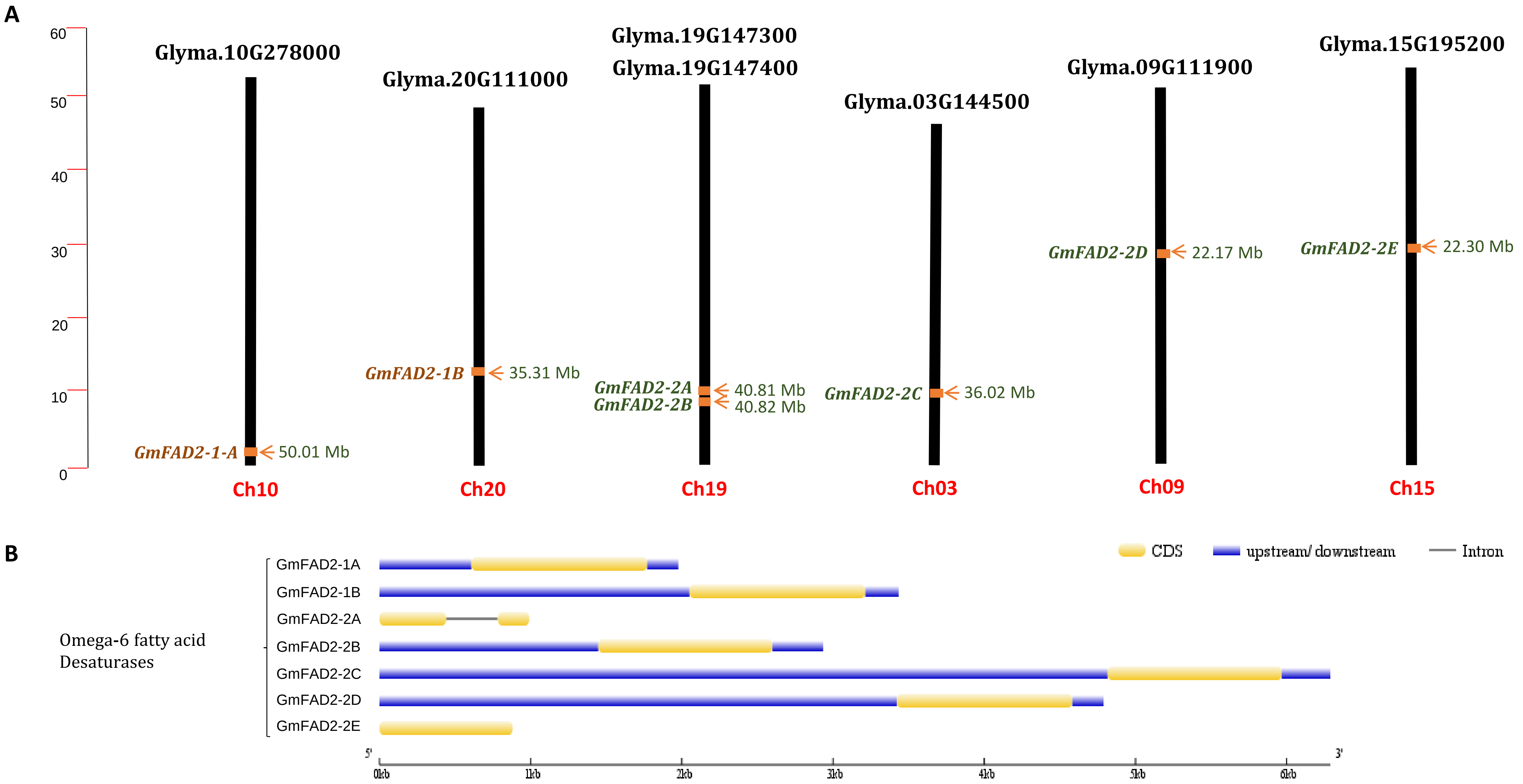

Figure S1. Physical positions and gene structures corresponding to the seven *GmFAD2*s are shown. (A) Six chromosomes carry the *GmFAD2* gene family. Glyma.10G278000 (Chr10: 50,013,484 – 50,015,460); Glyma.20G111000 (Chr20: 35,315,629 - 35,319,062); Glyma.19G147300 (Chr19: 40,814,864 - 40,815,855); Glyma.19G147400 (Chr19: 40,819,961 - 40,824,925); Glyma.03G144500 (Chr03: 36,014,623 - 36,020,910), Glyma.09G111900 (Chr09: 22,174,486 – 22,179,275), Glyma.15G195200 (Chr15: 22,308,021 – 22,308,902). **(B)** Gene structures of soybean fatty acid desaturases from three gene families. The structures of 19 soybean fatty acid desaturases genes were plotted with yellow boxes representing exons (coding DNA sequence, CDS) , black lines illustrating introns, and blue boxes indicating 5'-UTR and 3'-UTR regions. The size of gene structures could be measured by the scale in the unit of base pair (bp) at the bottom. The gene structure was drawn using the Gene Structure Display Server.

Table S1. Designed *GmFAD2-1A*, *GmFAD2-1B*, *GmFAD2-2A*, *GmFAD2-2B*, *GmFAD2-2C*, *GmFAD2-2D*, and *GmFAD2-2E* probes used for TILLING by Target Capture Sequencing.

Scaffold	Start	Stop	Glyma	#probes	bp covered	bp in region	% coverage	expected coverae with Flanking data
Chr10	50013364	50013926	Glyma.10G278000	12	560	562	0.996441	100
Chr10	50014094	50015460	Glyma.10G278000	32	1360	1366	0.995608	100
Chr20	35315510	35316655	Glyma.20G111000	26	1120	1145	0.978166	100
Chr20	35316764	35317641	Glyma.20G111000	19	840	877	0.957811	100
Chr20	35317682	35319063	Glyma.20G111000	32	1360	1381	0.984794	100
Chr19	40814864	40815305	Glyma.19G147300	9	440	441	0.997732	100
Chr19	40815527	40815975	Glyma.19G147300	9	440	448	0.982143	100
Chr19	40819962	40820319	Glyma.19G147400	6	320	357	0.896359	100
Chr19	40821993	40822850	Glyma.19G147400	17	760	857	0.886815	100
Chr19	40823429	40824926	Glyma.19G147400	35	1480	1497	0.988644	100
Chr03	36014506	36014942	Glyma.03G144500	7	360	436	0.825688	100
Chr03	36018896	36019188	Glyma.03G144500	5	280	292	0.958904	100
Chr03	36019431	36020913	Glyma.03G144500	35	1480	1482	0.998651	100
Chr09	22174485	22175865	Glyma.09G111900	32	1360	1380	0.985507	100
Chr09	22178991	22179274	Glyma.09G111900	5	280	283	0.989399	100
Chr15	22308021	22308902	Glyma.15G195200	20	880	881	0.998865	100

Table S2. Primers used for target Sanger sequencing.

Gene	Gene model	Primers	Primer Sequences	Products (bps)
GmFAD2-1A	Glyma.10G278000	FAD2-1A-Fw	CACATTCAGCAAAACAACTGA	1272
		FAD2-1A-Rv	TGTACTAATACATGACAAAAC	
GmFAD2-1B	Glyma.20G111000	FAD2-1B-Fw	GTATTAGACATTCAGCAACAAC	1273
		FAD2-1B-Rv	TTAAGTGATAAGTGACAAAAC	
GmFAD2-2A	Glyma.19G147300	FAD2-2A-Fw	GTTCATGAAAAGTGCATGGT	1114
		FAD2-2A-Rv	ATTCATCCTAAACCGTCACA	
GmFAD2-2B	Glyma.19G147400	FAD2-2B-Fw	TCGCTTTGATGAGAAACATT	1355
		FAD2-2B-Rv	ACAAAGGAAAGTTCAAACCA	
GmFAD2-2C	Glyma.03G144500	FAD2-2C-Fw	GTTCATTCTGATTCGGTGAG	1350
		FAD2-2C-Rv	CCCAATAAGCAACCATGATA	
GmFAD2-2D	Glyma.09G111900	FAD2-2D-Fw	TTGATGAGTTAGTTTTGCAATTA	1350
		FAD2-2D-Rv	TTTATAAGACCCATAAGGAACA	
GmFAD2-2E	Glyma.15G195200	FAD2-2E-Fw	TCACCATAGCCTTCTACCTC	1035
		FAD2-2E-Rv	AGCAACCATGCTACTAAACAG	

Table S3. Summary of the identified *cis-elements* at the promoter region (-2Kb upstream) of the translation start codon of *GmFAD2-1* and *GmFAD2-2* gene members showing an enrichment of the Arabidopsis homeobox protein domain within the seven *GmFAD2* members.

Matrix Family	p-value	Match Total	Common to #sequences	Glyma.10G278000	Glyma.20G111000	Glyma.19G147300	Glyma.19G147400	Glyma.03G144500	Glyma.09G111900	Glyma.15G195200
P$AHBP	0.01018	459	7	59	64	81	34	67	102	52
P$GTBX	0.005752	173	7	29	24	26	15	26	36	17
P$L1BX	0.020247	156	7	22	26	26	21	26	28	7
O$VTBP	0.048814	150	7	20	15	20	16	36	21	22
P$CCAF	0.023601	139	7	18	16	17	16	32	16	24
P$DOFF	0.018101	126	7	18	17	23	10	19	29	10
P$MYBL	0.002938	120	7	17	17	25	17	15	10	19
P$MYBS	0.01018	120	7	13	19	18	24	14	15	17
P$TCXF	0.043612	108	7	22	17	11	11	25	12	10
P$AHLF	0.024051	98	7	8	17	12	14	23	13	11
P$MIIG	0.014292	78	7	10	16	15	10	7	9	11
P$NTMF	0.038909	77	7	9	15	13	9	10	8	13
P$KAN1	0.036201	72	7	15	16	9	11	10	2	9
P$MADS	0.022292	67	7	15	8	10	4	9	9	12
P$SWNS	0.048801	59	7	5	10	13	7	11	6	7
P$NACF	0.031612	56	7	8	4	12	12	6	4	10
O$PTBP	0.003131	54	7	9	3	12	4	15	5	6
P$HEAT	0.044406	49	7	7	8	9	6	7	5	7
O$YTBP	0.000114	47	7	7	3	13	2	13	5	4
P$IBOX	0.048947	42	7	4	7	9	6	4	6	6
P$SUCB	0.033371	41	7	8	7	6	3	7	5	5
P$NCS1	0.037261	39	7	5	10	2	5	6	5	6
P$WBXF	0.041233	39	7	7	8	5	3	5	2	9
P$HMGF	0.003333	36	7	6	4	6	4	4	8	4
P$NACD	0.046665	36	7	4	3	9	6	2	6	6
P$MYCL	0.049085	35	7	6	9	3	9	4	3	1
P$SBPD	0.048161	32	7	5	9	3	1	4	5	5
P$LREM	0.040163	31	7	1	5	5	9	5	4	2
P$GLKF	0.046363	30	7	5	6	1	8	5	1	4
P$TELO	0.632334	29	6	0	4	3	2	10	3	7
P$TOEF	0.488227	29	6	5	0	8	1	6	3	6
P$TODS	0.037435	28	7	5	6	4	3	4	3	3
P$IDDF	0.012587	27	7	5	3	3	4	3	4	5
P$STKM	0.004879	27	7	2	5	5	4	9	1	1
P$OPAQ	0.048493	26	7	2	3	4	3	6	4	4
P$STKL	0.034083	25	7	3	2	6	2	4	5	3
P$SPF1	0.019852	24	7	2	5	3	5	3	5	1
P$C3HF	0.017845	23	7	6	4	1	2	5	4	1
P$GARP	0.033371	23	7	3	1	6	4	3	3	3

Table S3. Summary of the identified *cis-elements* at the promoter region (-2Kb upstream) of the translation start codon of *GmFAD2-1* and *GmFAD2-2* gene members showing an enrichment of the Arabidopsis homeobox protein domain within the seven *GmFAD2* members (continues).

Matrix Family	p-value	Match Total	Common to #sequences	Glyma.10G278000	Glyma.20G111000	Glyma.19G147300	Glyma.19G147400	Glyma.03G144500	Glyma.09G111900	Glyma.15G195200
P$SEF4	0.000194	23	7	2	1	6	3	5	2	4
P$GAGA	0.023343	22	6	0	4	1	9	2	3	3
P$GAPB	0.013498	22	7	5	2	4	2	5	3	1
P$JARE	0.023889	22	7	4	4	4	2	2	4	2
P$YABB	0.029828	22	7	3	2	3	2	2	3	7
P$GBOX	0.435033	20	6	5	3	3	6	2	0	1
P$LEGB	0.005707	20	7	3	1	4	1	4	3	4
P$CAAT	0.495715	18	5	0	2	4	3	0	4	5
P$NCS2	0.003906	18	7	3	3	1	3	1	6	1
P$WTBX	0.015833	18	7	2	6	1	1	4	2	2
P$ASRC	0.025369	16	7	1	5	2	2	1	2	3
P$TERE	0.055593	15	6	2	1	5	1	0	3	3
O$INRE	0.121698	14	6	0	2	3	3	2	2	2
P$AGP1	0.240687	14	6	0	1	3	4	2	2	2
P$ARID	0.007228	14	5	0	2	2	0	4	5	1
P$PCDR	0.010892	14	7	1	1	3	3	1	4	1
P$SCAP	0.294593	14	6	2	2	2	3	3	2	0
P$TCPF	0.31256	14	5	0	0	4	3	2	2	3
P$TGAF	0.68305	14	5	2	3	4	4	0	1	0
P$WOXF	0.012924	14	7	1	3	3	1	2	2	2
P$ABRE	0.422083	13	5	3	3	2	3	2	0	0
P$CNAC	0.332179	13	5	2	4	3	0	0	1	3
P$PSRE	0.01204	13	7	2	2	2	2	3	1	1
P$RAV5	0.004554	13	7	1	1	2	1	1	5	2
P$BRRE	0.612528	12	4	4	0	4	1	3	0	0
P$FBHF	0.004816	12	6	1	1	0	2	2	4	2
P$NIGS	0.083845	12	6	2	2	2	0	1	1	4
P$ZFAT	0.017158	12	7	1	2	1	1	2	2	3
P$AP2L	0.464113	10	5	0	2	2	1	1	0	4
P$DREB	0.933442	10	5	2	2	0	0	4	1	1
P$EPFF	0.210486	10	5	2	4	1	2	0	1	0
P$FLO2	0.162584	10	6	2	1	2	1	2	0	2
P$GAZL	0.336167	10	5	2	0	1	1	3	3	0
P$TEFB	0.052654	10	6	1	1	2	1	0	3	2
P$EINL	0.285931	9	4	1	5	2	0	1	0	0
P$PSPE	0.493438	9	4	2	2	2	3	0	0	0
P$ROOT	0.187627	9	5	2	0	4	1	0	1	1
P$CARM	0.002261	8	7	1	1	1	1	2	1	1
P$MSAE	0.860461	8	5	1	3	0	1	1	0	2

Table S3. Summary of the identified *cis-elements* at the promoter region (-2Kb upstream) of the translation start codon of *GmFAD2-1* and *GmFAD2-2* gene members showing an enrichment of the Arabidopsis homeobox protein domain within the seven *GmFAD2* members (continues).

Matrix Family	p-value	Match Total	Common to #sequences	Glyma.10G278000	Glyma.20G111000	Glyma.19G147300	Glyma.19G147400	Glyma.03G144500	Glyma.09G111900	Glyma.15G195200
P$PNRE	0.803438	8	4	1	3	0	0	1	3	0
P$FRSF	0.217304	7	2	0	1	0	6	0	0	0
P$PAHD	0.063272	7	5	2	0	1	1	1	0	2
P$REMF	0.257143	7	5	1	1	1	2	0	2	0
P$RKDS	0.259472	7	4	1	2	0	0	0	1	3
P$AREF	0.037029	6	5	1	1	1	0	2	0	1
P$CGCG	0.94961	6	2	0	3	0	3	0	0	0
P$SRSF	0.242523	6	1	0	0	6	0	0	0	0
P$BBZF	0.00679	5	5	1	1	1	0	1	1	0
P$FORC	0.273173	5	3	0	2	0	1	2	0	0
P$LBDF	0.991552	5	2	0	0	0	4	1	0	0
P$LFYB	0.214942	5	4	0	1	0	1	1	0	2
P$OCSE	0.199846	5	4	1	0	1	1	0	0	2
P$URNA	0.249845	5	3	0	1	3	0	1	0	0
P$CE1F	0.198201	4	4	1	1	0	1	0	0	1
P$GCCF	0.878634	4	3	1	0	0	2	1	0	0
P$SEF3	0.615166	4	2	0	1	0	3	0	0	0
P$DPBF	0.437001	3	2	2	0	0	0	1	0	0
P$PALA	0.124814	3	3	0	0	1	1	1	0	0
P$TDTF	0.81948	3	2	0	0	0	2	0	1	0
P$VRES	0.812229	3	2	0	1	0	0	0	2	0
O$MTEN	0.74333	2	2	0	1	0	0	1	0	0
O$TF3A	0.659894	2	2	0	1	0	0	0	0	1
P$ARF3	0.962997	2	2	0	1	0	1	0	0	0
P$ERSE	0.562499	2	2	0	0	0	1	1	0	0
P$GRSF	0.916971	2	2	0	1	1	0	0	0	0
P$MGSA	0.951258	2	2	0	0	1	0	0	1	0
P$SAMM	0.29964	2	1	0	0	0	2	0	0	0
P$TRIH	0.429165	2	1	0	0	0	2	0	0	0
O$TELO	0.253087	1	1	0	1	0	0	0	0	0
P$CDC5	0.1095	1	1	0	0	0	1	0	0	0
P$E2FF	0.408546	1	1	0	0	1	0	0	0	0
P$EREF	0.410726	1	1	1	0	0	0	0	0	0
P$HOCT	0.218985	1	1	0	0	0	1	0	0	0
P$LICM	0.314588	1	1	0	0	1	0	0	0	0
P$MYCS	0.151131	1	1	0	0	0	1	0	0	0
P$NCS3	0.118303	1	1	0	0	0	0	0	0	1
P$PREM	0.265962	1	1	1	0	0	0	0	0	0
P$SALT	0.305401	1	1	0	0	0	0	0	0	1
P$SURE	0.001506	1	1	1	0	0	0	0	0	0
